# Plutonium age dating (production date measurement) by inductively coupled plasma mass spectrometry

**DOI:** 10.1007/s10967-015-4418-5

**Published:** 2015-09-04

**Authors:** Zsolt Varga, Adrian Nicholl, Maria Wallenius, Klaus Mayer

**Affiliations:** European Commission, Joint Research Centre (JRC), Institute for Transuranium Elements, Postfach 2340, 76125 Karlsruhe, Germany

**Keywords:** Age dating, Radiochronometry, Plutonium, Nuclear safeguards, Nuclear forensics

## Abstract

This paper describes rapid methods for the determination of the production date (age dating) of plutonium (Pu) materials by inductively coupled plasma mass spectrometry (ICP-MS) for nuclear forensic and safeguards purposes. One of the presented methods is a rapid, direct measurement without chemical separation using ^235^U/^239^Pu and ^236^U/^240^Pu chronometers. The other method comprises a straightforward extraction chromatographic separation, followed by ICP-MS measurement for the ^234^U/^238^Pu, ^235^U/^239^Pu, ^236^U/^240^Pu and ^238^U/^242^Pu chronometers. Age dating results of two plutonium certified reference materials (SRM 946 and 947, currently distributed as NBL CRM 136 and 137) are in good agreement with the archive purification dates.

## Introduction

The international safeguards system implemented by the International Atomic Energy Agency (IAEA) has been set up to in order to prevent the malicious use of nuclear materials, and to verify the correctness and completeness of states’ declarations about the nuclear-related activities and nuclear material accountancy [1]. However, if such materials are diverted and then interdicted, detailed investigation is required to identify the possible origin, intended use and hazard related to the material. Such analyses, which are referred to as *nuclear forensics*, involve the comprehensive physical, chemical and isotopic analyses of the nuclear material as well as the interpretation of the measured parameters along with additional information on the material in question [2, 3]. Several characteristic parameters (so-called *signatures*) of the material can be used for this purpose, such as isotopic composition of U, Pb or Sr, elemental impurities, trace-level radionuclide content, crystal structure or anionic residues [2–6]. Besides these parameters, the elapsed time since the last chemical purification of the material (commonly referred to as the “age” of the material) can also be measured for radioactive and nuclear materials [2, 7, 8]. This unique possibility is based on the presence of radionuclides and their radioactive decay: during its production, the radioactive material is chemically purified from the impurities, including also its radioactive decay products. After the chemical separation of a radionuclide, its radioactive progenies start to grow-in into the material. The theoretical amount of daughter nuclide formed by the decay can be calculated by the use of the radioactive decay equations (Bateman equations) [9]. The ratio of the daughter nuclide amount relative to the amount of its parent nuclide can be calculated as follows:

1 $$\frac{{N_{\text{daughter}} }}{{N_{\text{parent}} }} = \frac{{\lambda_{\text{parent}} }}{{\lambda_{\text{daughter}} - \lambda_{\text{parent}} }}\left( {e^{{ - \lambda_{\text{parent}} t}} - e^{{ - \lambda_{\text{daughter}} t}} } \right) + \frac{{N_{\text{daughter}}^{ 0} }}{{N_{\text{parent}} }}e^{{ - \lambda_{\text{daughter}} t}}$$

where *N*_daughter_*/N*_parent_ is the amount (atom) ratio of the daughter and parent nuclides in the sample, *λ*_daughter_ and *λ*_parent_ are the decay constants of daughter and parent nuclides, respectively, $N_{\text{daughter}}^{ 0}$ is the residual daughter nuclide after the chemical separation, and *t* is the elapsed time since the separation of the radionuclides. The daughter-to-parent ratio (*N*_daughter_*/N*_parent_) is often referred to as a *chronometer*, while the elapsed time (*t*) is called the *age* of the material.

The age dating model assumes that the sample behaves as a closed system, meaning that there is no loss or increase for either the parent nuclide or for the decay products after production. If the initial concentration of the daughter nuclide is zero after the last chemical separation (i.e. the separation was complete, $N_{\text{daughter}}^{ 0}$ equals to zero), and the amount ratio of the parent and daughter nuclide is measured, the elapsed time (*t*) can be calculated as follows:

2 $$t = \frac{1}{{\lambda_{\text{parent}} - \lambda_{\text{daughter}} }}\ln \left( {1 - \frac{{N_{\text{daughter}} }}{{N_{\text{parent}} }} \cdot \frac{{\lambda_{\text{daughter}} - \lambda_{\text{parent}} }}{{\lambda_{\text{parent}} }}} \right)$$

This age value and the respective production date can help either to identify the origin of the questioned unknown sample or to verify the source of the starting nuclear material used for production. By determining the elapsed time (*t*) and knowing the reference date of the daughter-to-parent ratio measurement, the separation date of the parent nuclide can be calculated, which is referred to as the production date of the material. Note that this model date corresponds to the time when the parent nuclide was chemically separated from its progeny in a production process step, thus different chronometers may give different ages reflecting different chemical process steps. In contrast to most other characteristic parameters used in nuclear safeguards or forensics, the production date of the material is a predictive signature, thus it does not require comparison samples for origin assessment (i.e. it is a self-explaining parameter). This feature makes the production date one of the most prominent signatures in nuclear forensics.

Although the age dating for uranium materials has been relatively widely studied and applied, plutonium age dating is often a more difficult task mainly due to radiation protection reasons and the more cumbersome handling of Pu. Determination of the trace-level U and Am is also challenging, especially when it is performed in a glove-box in a nuclear environment. The age of a Pu material can determined using several chronometers, such as ^241^Am/^241^Pu, ^234^U/^238^Pu, ^235^U/^239^Pu, ^236^U/^240^Pu or ^238^U/^242^Pu. The ^241^Am/^241^Pu ratio can also be determined using non-destructive gamma spectrometry [10–13], which is well-established and implemented in several commercially available software codes. However, it requires higher amount of sample compared to the destructive techniques. Out of the destructive methods alpha spectrometry and mass spectrometric techniques including thermal ionisation mass spectrometry (TIMS) [10, 12, 14] or inductively coupled plasma mass spectrometry (ICP-MS) [12, 13, 15], and the combination of alpha spectrometry and TIMS [15–17] have been reported. In general, TIMS analysis requires more material for age dating (>μg amount of Pu), while for ICP-MS less material is needed and it is less susceptible to the amount of the residual dissolved solid in the analysed sample aliquot after chemical separation. The chemical separation required for the destructive techniques comprises ion exchange [10, 17] or extraction chromatographic separation [10, 11, 13, 15, 17]. In most cases the chemical separation is tedious and lengthy, and involves a series of separation steps to achieve appropriate purity. Often not all chronometers can be used due to the elevated initial U content in the sample (incomplete separation) or potential U contamination [15, 17]. For the age dating of individual Pu particles secondary ionisation mass spectrometry (SIMS) [18], ICP-MS with or without chemical separation [19–21], and wavelength dispersive X-ray spectrometry (WDX) coupled to a scanning electron microscope (SEM), supplemented with TIMS measurement have been reported [22]. As no reference material is available for Pu age dating, it is very difficult to validate the methods, especially for U/Pu chronometers, since U is present at trace-level. One possible approach for validation is the age dating of well-characterised Pu standard materials certified for other characteristics, and to compare them with the available information on the archive production history of the material and with the other measured results reported in the literature.

The aim of the present work is to develop two methods for the age dating of Pu material by ICP-MS measurement. One of the methods is a rapid, direct ICP-MS measurement of the ^235^U/^239^Pu and ^236^U/^240^Pu chronometers, which does not require chemical separation of the material. The other method involves a simple and straightforward chemical separation of the U daughter products from the Pu material, followed by an ICP-MS measurement. The method with chemical separation was planned in such a way that it could be extended for the ^241^Am/^241^Pu chronometer in the future.

## Experimental

### Reagents and materials

All labware was thoroughly cleaned before use. Nitric acid was Suprapur grade (Merck, Darmstadt, Germany), which was further purified by subboiled distillation (AHF analysentechnik AG, Germany). For dilutions ultrapure water was used (Elga LabWater, Celle, Germany). Purum grade hydroxylamine nitrate solution (18 % NH_2_OH·HNO_3_ in H_2_O) and analytical grade NaNO_2_ were purchased from Sigma-Aldrich (Steinheim, Germany). A ^233^U and ^242^Pu isotopic standards were used to spike the samples for the U concentration measurements. The ^233^U concentration in the spike was calibrated against EC NRM 101 U metal by thermal ionization mass spectrometry (TIMS), while the ^242^Pu spike was IRMM-085 standard from the Institute for Reference Materials and Measurements (Geel, Belgium). Nominally 1 % enriched uranium U-010 standard reference material from National Bureau of Standards (NBS, USA) was used to correct for instrumental mass discrimination. IRMM-185 (certified *n*(^235^U)/*n*(^238^U) is (2.00552 ± 0.00060) × 10^−2^) isotopic reference material was used to check the accuracy of the U isotope ratio measurements. TEVA extraction chromatographic resin (50–100 μm particle size, active component: aliphatic quaternary amine) supplied by Triskem International (Bruz, France) was used for the chemical separation. 0.4 mL of the TEVA resin was placed in plastic Bio-Rad column holders (diameter: 6 mm, length: 14 mm) and was covered with a porous Teflon frit (Reichelt Chemietechnik Heidelberg, Germany) to avoid resin mixing. Before use, the column was cleaned with 1 mL of 0.02 M HF/0.02 M HNO_3_ followed by conditioning with 4 mL 3 M HNO_3_.

### Analysed plutonium reference materials

The measured Pu samples in the present study are the NBS 946 and 947 certified reference materials, certified for Pu isotopic composition. The Pu is of reactor-grade isotopic composition. The standards are currently distributed as NBL CRM 136 and CRM 137, respectively, by New Brunswick Laboratory (NBL), Argonne, USA. The samples are in the form of plutonium sulphate tetrahydrate. According to the NBL archives the purification of the Pu sulphate crystals of NBS 946 and NBS 947 were completed in January 1971 and in December 1970, respectively. Note that these purification dates are different than the release of the certificate, which occurred later. We have no further information on degree of purification, and it is assumed that the U and Am separation from Pu was complete at the time of production

For the analysis approximately 25 mg Pu standard was dissolved with 8 mL 8 M HNO_3_ solution in a perfluoroalkoxy alkane (PFA) screw cap vial. The obtained stock solution was diluted to about 5 μg g^−1^ Pu concentration volumetrically with 3 M HNO_3_. This diluted working solution was used afterwards for the age dating measurements.

### Instrumentation and analytical measurements

The Pu and U isotopic measurements were carried out using a double-focusing magnetic sector ICP-MS equipped with a single electron multiplier (Element 2, Thermo Electron Corp., Bremen, Germany). The ICP-MS instrument is attached to a nuclearized glove box in order to handle Pu. All measurements were carried out in low resolution mode (*R* = 300) using a low-flow micro-concentric nebulizer operated in a self-aspirating mode (flow rate was approximately 50 μL min^−1^) in combination with a stable introduction system (SIS) quartz glass spray chamber. The measured isotope ratios obtained by ICP-MS were corrected for instrumental mass bias using linear correction. All dilutions and spike additions for the isotope dilution analysis were done gravimetrically. The solution weights were obtained as the difference of the weight of the sample in the measurement vials and the tare vial weights for each step.

### Age measurement by direct ICP-MS measurement

As the typical U/Pu mass ratio in Pu samples with an age of 40 years is about 10^−3^, it is possible to measure the ^235^U/^239^Pu and ^236^U/^240^Pu ratios by ICP-MS without the chemical separation of U. The measurement of ^234^U/^238^Pu and ^238^U/^242^Pu chronometers cannot be accomplished due to the possible interference from ^238^U and ^238^Pu, respectively. Similarly, the direct measurement of the ^241^Am/^241^Pu ratio is hindered by the almost identical masses of ^241^Am and ^241^Pu.

**Fig. 1:**
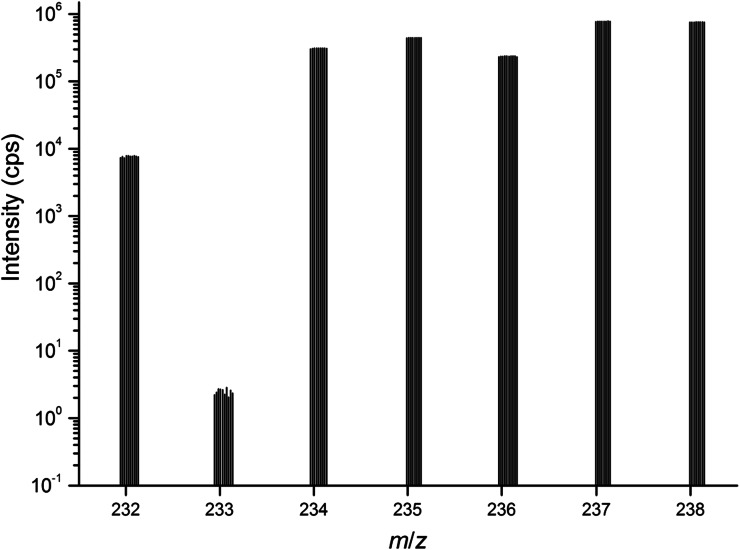
Measured spectrum of the unspiked SRM 946 sample using the direct method. Note that for the isotope ratio evaluation the peak centroid of the isotopes of interest (*flat-top peak*) was selected

In order to perform the direct measurement, an aliquot of the diluted working solution was gravimetrically weighed into a pre-cleaned polyethylene (PE) vial, and mixed with a weighed aliquot of the ^233^U spike. The sample was thoroughly homogenized. In parallel to that an unspiked aliquot of the sample as well as a blank sample was prepared. The Pu concentration in the spiked and unspiked samples was approximately 150 ng g^−1^, and the sample was analysed by ICP-MS without additional dilution. The measurements were done in duplicates. The date of the measurement was chosen as reference date. An example of the measured spectrum is shown in Fig. 1.

Other aliquots of the diluted working solutions were taken to determine the ^239^Pu and ^240^Pu amount content by isotope dilution ICP-MS using ^242^Pu spike.

### Age measurement by ICP-MS after chemical separation

**Fig. 2:**
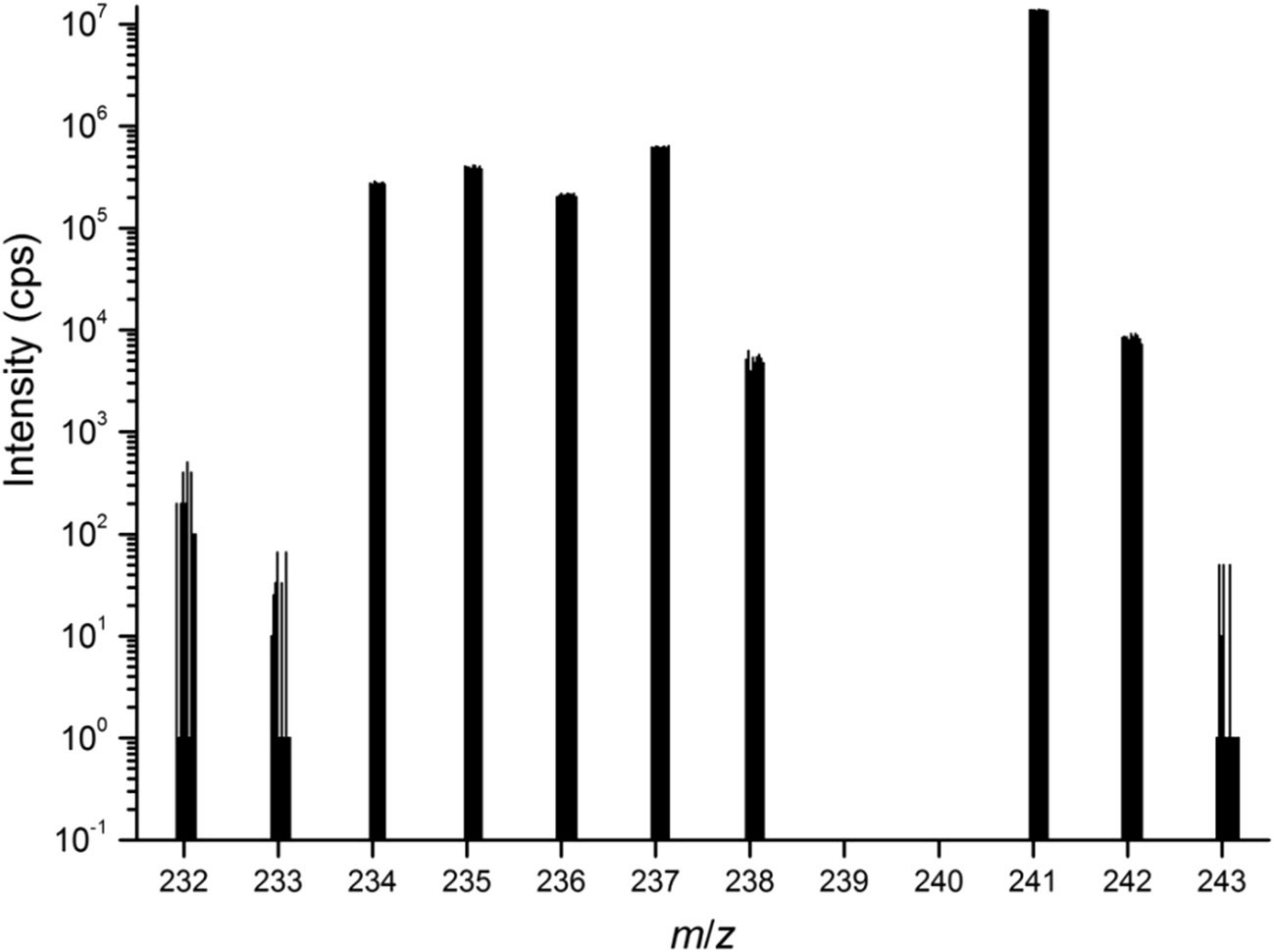
Measured spectrum of the unspiked SRM 946 sample after the chemical separation. Note that the signals at *m*/*z* = 239 and *m*/*z* = 240 were omitted due to the high intensity (about 2 × 10^6^ and 2 × 10^5^ cps, respectively), and for the isotope ratio evaluation the peak centroid of the isotopes of interest was selected

In order to minimize the sample manipulation and the generated waste, extraction chromatography was selected to separate Pu from U. A sample aliquot with approximately 1 μg of Pu (200 μL of the diluted working solution) was spiked with ^233^U spike gravimetrically. The solution was mixed with 1 mL 3 M HNO_3_/0.02 M NH_2_OH·HNO_3_. The NH_2_OH·HNO_3_ serves to adjust the oxidation state of Pu to Pu(III). After a few minutes waiting 60 μL 3 M NaNO_2_ was added to the sample at ambient temperature, which oxidizes the Pu to Pu(IV) oxidation state. Heating of the sample can accelerate the reactions and help to reduce bubble formation during the column separation. Under such conditions Pu(IV) and Th(IV) retains strongly on the TEVA resin, while U and Am have little affinity to the resin [23]. After loading the solution on the TEVA resin the vial and the column were washed with 3 mL 3 M HNO_3_. The load and wash solution were collected together in a 10-mL PE vial, resulting in about 4.5 mL solution. The time of the chemical separation was registered as the reference date for age dating. This sample was measured by ICP-MS after a 10-fold dilution with 2 % HNO_3_. An example of the measured spectrum is shown in Fig. 2. Note that ^237^Np and ^241^Am can also be recovered with the proposed sample preparation. In parallel to the spiked sample, a blank and an aliquot of the unspiked sample were also subjected to the chemical separation and a forthcoming analysis by ICP-MS. The Pu of the unspiked sample was separately eluted from the TEVA column with 3 mL 1 M HNO_3_/0.02 M NH_2_OH·HNO_3_ in a PE vial to measure the Pu isotopic composition of the sample. The separations were done in duplicates. The separation could be performed within a few hours, with an estimated U recovery of higher than 95 % and a Pu separation factor higher than 200. Such a separation factor is sufficient for higher burn-up Pu samples.

Other aliquots of the starting diluted working solutions were also taken to determine the ^239^Pu amount content by isotope dilution ICP-MS using ^242^Pu spike. The spiked samples were also subjected to the chemical separation. The *n*(^238^Pu), *n*(^239^Pu), *n*(^240^Pu) and *n*(^242^Pu) amount contents needed for the age dating calculations were calculated using the measured Pu isotopic composition of the separated unspiked Pu sample and the ^239^Pu amount content obtained by isotope dilution ICP-MS.

### Data evaluation

Concentrations of isotopes of interest necessary for the production date calculation were experimentally determined according to the isotope dilution method. The measured amount contents of the required U and Pu isotopes were used to calculate the (model) age of the material according to Eq. (2). The ^238^U content in the chemically separated sample was calculated by the mathematical correction for the ^238^Pu at *m*/*z* = 238 by subtracting the ^238^Pu contribution from the measured signal using the separately measured *n*(^238^Pu)/*n*(^242^Pu) ratio of the separated, unspiked Pu sample. The overall uncertainties were calculated taking into account the uncertainty of the weight measurements, spike concentrations, measured isotope ratios, relative atomic masses and half-lives according to ISO/BIPM guide [24]. The given uncertainties in the present work are expanded uncertainties with a coverage factor of *k* = 2. The age calculations were performed with commercially available software, GUM Workbench [25]. The Decay Data Evaluation Project (DDEP) recommended half-lives were used for the age calculations. The used half-lives of ^234^U, ^235^U, ^236^U, ^238^U, ^238^Pu, ^239^Pu, ^240^Pu and ^242^Pu were 2.455 × 10^6^ ± 600, 7.04 × 10^8^ ± 1 × 10^6^, 2.343 × 10^7^ ± 4 × 10^4^, 4.468 × 10^9^ ± 5 × 10^6^, 87.74 ± 0.03, 24100 ± 11, 6561 ± 7 and 3.73 × 10^5^ ± 3 × 10^3^ years (*k* = 1), respectively [26]. The dominant uncertainty components were the measured isotope ratios in the spiked sample and the spike concentrations, except for the ^238^Pu and ^238^U measurements, where the dominant uncertainty contributors were the blank level and the ^238^Pu mathematical correction, respectively.

## Discussion and results

### Age results of the plutonium certified reference materials

The measured age results of the Pu certified reference materials are summarized in Table 1. The results are given relative to a common reference date of 11 March 2015 (date of the plutonium separation). The results obtained with and without the chemical separation agree well within measurement uncertainty. Also the ages obtained by the different chronometers give identical results (concordant ages). The achievable uncertainties by the direct method for such old materials are comparable to the results measured after chemical separation. The ^234^U/^238^Pu values have higher uncertainty due to the higher uncertainty in the lower ^238^Pu signal and the elevated background at *m*/*z* = 238. The ^238^U/^242^Pu age results have far too high uncertainty compared to the other chronometers: this is due to the lower amount of ^238^U compared to the other U decay products and its difficult measurement by mass spectrometry (interference from the ^238^Pu, elevated background at *m*/*z* = 238 and high risk of ^238^U contamination). In our separation and measurement scheme the residual ^238^Pu signal contribution was in the same order of magnitude as the progeny ^238^U signal.

**Table 1:** Measured age dating results of the plutonium certified reference materials using the different chronometers

	Chronometers			
	^234^U/^238^Pu	^235^U/^239^Pu	^236^U/^240^Pu	^238^U/^242^Pu
SRM946				
Age determined by chemical separation (years)	44.4 ± 1.2	44.56 ± 0.46	44.07 ± 0.60	150 ± 110
Age measured by direct analysis (years)	NA	44.46 ± 0.47	43.77 ± 0.61	NA
SRM947				
Age determined by chemical separation (years)	42.8 ± 2.5	44.15 ± 0.11	44.36 ± 0.18	76 ± 55
Age determined by direct analysis (years)	NA	44.57 ± 0.73	44.36 ± 0.19	NA

Uncertainties are expressed as expanded uncertainties (*k* = 2) (reference date: 11 March 2015)

### Calculated production dates

Knowing the determined age of the material and the time of the chemical separation, the absolute production date of the samples can be calculated (Table 2). The ^238^U/^242^Pu age results with too large uncertainty were left out for ease of comparison. The measured production dates of the SRM 946 and 947 samples agree well with the known, archive dates of production (January 1971 and in December 1970, respectively). This agreement implies that the U separation from the Pu material was complete during the purification step.

**Table 2:** Calculated production date results of the plutonium certified reference materials for the different chronometers

	Chronometers		
	^234^U/^238^Pu	^235^U/^239^Pu	^236^U/^240^Pu
SRM946			
Production date by chemical separation	October 1970 ± 1.2 years	August 1970 ± 6 months	February 1971 ± 7 months
Production date determined by direct analysis	NA	September 1970 ± 6 months	June 1971 ± 7 months
SRM947			
Production date by chemical separation	May 1972 ± 2.5 years	15 January 1971 ± 1 month	30 October 1970 ± 2 months
Production date determined by direct analysis	NA	August 1970 ± 9 months	30 October 1970 ± 2 months

Uncertainties are expressed as expanded uncertainties (*k* = 2)

**Fig. 3:**
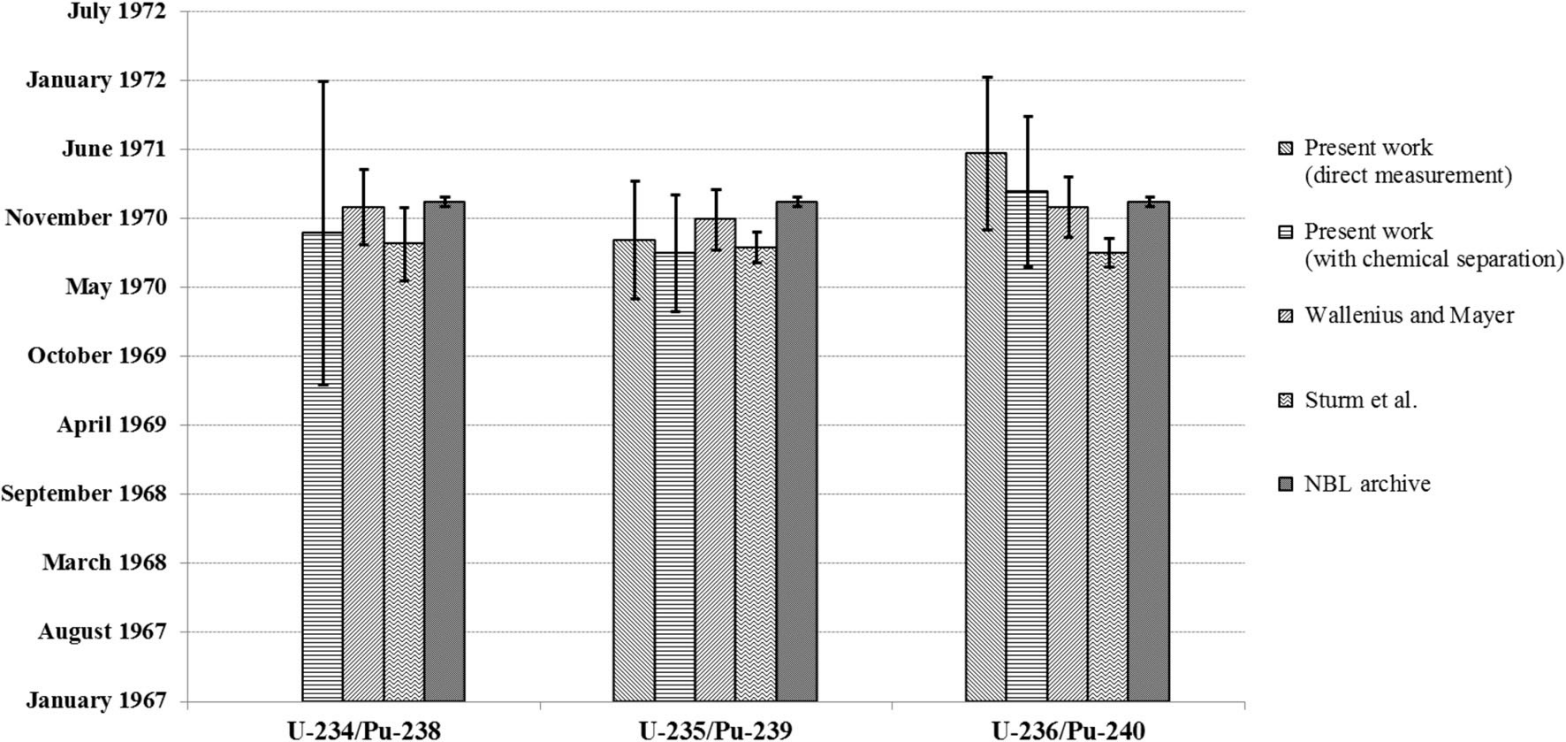
Comparison of the measured and reported production dates for SRM 946 standard [10, 17]. Uncertainties are expressed as reported (either as 95 % confidence level or as expanded uncertainties at *k* = 2)

The obtained absolute production dates allow for the comparison with previously reported results in the literature (Fig. 3) [10, 17]. For ease of comparison only the values for SRM 946 are shown here, which has been studied more extensively. The reported production dates were calculated as the difference of the reference date and the measured ages. The uncertainties shown in Fig. 3 are as reported, either as 95 % confidence level or as expanded uncertainties at *k* = 2. As the U/Pu ages were measured after two different sample preparation techniques by Wallenius and Mayer [10], the average results are given. As the exact date of the purification by day was not available from the NBL archive, and it is given only as January 1971, an uncertainty of half a month was assigned to this date.

The reported production dates are in relatively good agreement with one another and also with the known, archive purification date. Note that the production dates based on different chronometers reported by the same groups are in better agreement than the absolute production dates reported by the different groups. This fact may be related to the differences in the analytical results/calculations or to the different definitions in the reference date.

## Conclusions

Two rapid measurement methods have been developed for the determination of the age and the respective production date of plutonium materials by ICP-MS for nuclear forensics and safeguards. One of the methods is based on the rapid, direct measurement of ^235^U/^239^Pu and ^236^U/^240^Pu chronometers. Depending on the age and isotopic composition of the sample, it allows the fast and relatively precise (1–2 %) age determination down to sub-nanogram amount of Pu samples. The other method, which involves the chemical separation of U, is particularly suited for age dating of freshly separated Pu materials with low amount of U decay products, for Pu samples with low ^240^Pu abundance (e.g. weapons grade Pu) and it also allows using the ^234^U/^238^Pu chronometer. The minimum amount of sample needed for the analysis is in the sub-nanogram range. The method using the chemical separation can also be extended to the measurement of ^241^Am/^241^Pu and ^237^Np/^241^Am chronometers. The methods have been applied for the age dating measurement of two Pu isotopic reference materials (SRM 946 and 947), and the experimentally determined production dates are in good agreement with the known, archive date of material purification. Small discrepancies between the literature values have been found for the SRM 946, which may be related to analytical measurement issues or the differences in the age calculations (e.g. use of different half-life values).
